# Rapid response of leaf photosynthesis in two fern species *Pteridium aquilinum* and *Thelypteris dentata* to changes in CO_2_ measured by tunable diode laser absorption spectroscopy

**DOI:** 10.1007/s10265-015-0736-5

**Published:** 2015-06-03

**Authors:** Keisuke Nishida, Naomi Kodama, Seiichiro Yonemura, Yuko T. Hanba

**Affiliations:** The Graduate School of Science, Kyoto Institute of Technology, Matsugasaki, Sakyo-ku, Kyoto, 606-8585 Japan; Agro-Meteorology Division, National Institute for Agro-Environmental Sciences, 3-1-3 Kannondai, Tsukuba, 305-8604 Japan; Swiss Federal Institute for Forest, Snow and Landscape Research (WSL), Zuricherstrasse 111, 8903 Birmensdorf, Switzerland; School of Human Science and Environment, Hyogo University, 1-1-12 Shinzaike-honcho, Himeji, 607-0092 Japan; Department of Applied Biology, Kyoto Institute of Technology, Matsugasaki, Sakyo-ku, Kyoto, 606-8585 Japan

**Keywords:** CO_2_ response, Ferns, Mesophyll conductance, Pteridophytes, Photosynthesis

## Abstract

We investigated stomatal conductance (*g*_s_) and mesophyll conductance (*g*_m_) in response to atmospheric CO_2_ concentration [CO_2_] in two primitive land plants, the fern species *Pteridium aquilinum* and *Thelypteris dentata*, using the concurrent measurement of leaf gas exchange and carbon isotope discrimination. [CO_2_] was initially decreased from 400 to 200 μmol mol^−1^, and then increased from 200 to 700 μmol mol^−1^, and finally decreased from 700 to 400 μmol mol^−1^. Analysis by tunable diode laser absorption spectroscopy (TDLAS) revealed a rapid and continuous response in *g*_m_ within a few minutes. In most cases, both ferns showed rapid and significant responses of *g*_m_ to changes in [CO_2_]. The largest changes (quote % decrease) were obtained when [CO_2_] was decreased from 400 to 200 μmol mol^−1^. This is in contrast to angiosperms where an increase in *g*_m_ is commonly observed at low [CO_2_]. Similarly, fern species observed little or no response of *g*_s_ to changes in [CO_2_] whereas, a concomitant decline of *g*_m_ and *g*_s_ with [CO_2_] is often reported in angiosperms. Together, these results suggest that regulation of *g*_m_ to [CO_2_] may differ between angiosperms and ferns.

## Introduction

Atmospheric CO_2_ is a substrate for leaf photosynthesis in land plants, and thus CO_2_ availability at the carboxylation site is one of the most important limiting factors for leaf photosynthesis. In the process of leaf photosynthesis in C_3_ land plants, CO_2_ diffuses from the atmosphere through stomata, intercellular air spaces, and the leaf mesophyll to the site of carboxylation in the chloroplasts. CO_2_ concentration in the chloroplast is lower than that in the atmosphere because of significant resistance to CO_2_ diffusion through this diffusional pathway, i.e., limitations in CO_2_ diffusion strongly reduce leaf photosynthesis. There are two major CO_2_ diffusional limitations; CO_2_ conductance though stomata, *g*_s_, and that from substomatal cavities to the chloroplast, termed *g*_m_.

Atmospheric CO_2_ levels have changed substantially over the evolutionary history of land plants. It is estimated that atmospheric CO_2_ levels were approximately 10 times higher than the present when land plants started to evolve 360–480 million years ago (Royer et al. 2004). Ferns are a major component of the fossil flora, and although they are primitive, non-seed plants, they are closely related to seed plants (Pryer et al. 2001). Atmospheric CO_2_ levels fell abruptly during the Cretaceous period (Kuypers et al. 1999), which coincides with a major diversification in the fern group (Pryer et al. 2004). On the other hand, angiosperms, which are currently the dominant group of seed plants, emerged during a period when atmospheric CO_2_ level was only two- to three-fold higher than the present (Haworth et al. 2011). This implies that ferns and angiosperms evolved under different selection pressures, which may have resulted in different mechanisms of CO_2_ diffusion between these two plant groups (Carriquí et al. 2015). Changes in the mechanisms of CO_2_ diffusion in plant evolutionary history have been suggested because stomatal frequency in fossil plants, which strongly affects *g*_s_, has been shown to track changes in atmospheric CO_2_ level (Woodward 1998). As such, it’s suggested that stomatal function developed to enhance CO_2_ diffusion to cope with decreases in CO_2_ level. However, changes in *g*_m_ in land plant history cannot be determined through similar anatomical imprints in the fossil record. Although the difference in *g*_m_ is possibly still partially reflected in extant plants of angiosperms and ferns. Leaf mesophyll anatomy affecting *g*_m_, including chloroplast surface area facing the intercellular airspaces and cell wall thickness, could have changed from ferns to angiosperms (Carriquí et al. 2015), which may be affected by the decrease in atmospheric CO_2_ level. A comparison of CO_2_ diffusional limitations in extant ferns with extant angiosperms could provide crucial information to estimate how photosynthesis traits have evolved in land plants. If atmospheric CO_2_ levels can influence selection pressure, phylogenetically distant fern groups may also vary in internal morphology and *g*_m_.

In the present atmospheric CO_2_ conditions, fern species have much lower photosynthetic capacity than angiosperms (Wright et al. 2005). In ferns, both *g*_s_ and *g*_m_ are lower than in angiosperms. A lower *g*_m_ is suggested to be the major mechanism underlying the lower photosynthetic capacity of fern species (Carriquí et al. 2015). However, there are only three published determinations of *g*_m_ of fern species to the best of our knowledge (Carriquí et al. 2015; Gago et al. 2013; Volkova et al. 2009). Anatomical and physiological mechanisms underlying the low *g*_m_ of fern species still remain to be confirmed.

The response of *g*_s_ to atmospheric CO_2_ concentration [CO_2_] is different between angiosperms and ferns. Extensive studies on angiosperms have shown that *g*_s_ typically increases with a decrease in [CO_2_] (e.g., Brodribb et al. 2009; Messinger et al. 2006). However, three ferns, *Osmunda regalis*, *Blechnum gibbum* and *Nephrolepis exaltata*, showed small responses to changes in [CO_2_] (Gago et al. 2013). The averaged *g*_s_ for six ferns and lycophytes showed no response to an increase in [CO_2_] above ambient, while they showed a slight increase with a decrease in [CO_2_] (Brodribb et al. 2009). Studies for the response of *g*_m_ to [CO_2_] are limited compared with *g*_s_ in angiosperms, and to the best of our knowledge, there is only one published study on the response of *g*_m_ to [CO_2_] in fern species (Gago et al. 2013). For angiosperms, there is conflicting evidence as to how *g*_m_ responds to [CO_2_]. Some studies reported insignificant effects of [CO_2_] on *g*_m_ (Harley et al. 1992; Tazoe et al. 2009), whereas other studies reported a decline in *g*_m_ at high [CO_2_] (Bunce 2010; Douthe et al. 2011; Hassiotou et al. 2009; Loreto et al. 1992, Tazoe et al. 2011), or showed curved responses to changes in [CO_2_] (Flexas et al. 2007; Vrábl et al. 2009). Gago et al. (2013) obtained a curved response in *g*_m_ with changes in [CO_2_] for three fern species. However, the observed decline in *g*_m_ at low [CO_2_] in angiosperms and ferns (sub-stomatal CO_2_ concentration, *C*_i_ < 50 µmol mol^−1^) may be an artifact related to partially photorespired CO_2_ (Tholen et al. 2012). Furthermore, the chlorophyll fluorescence technique used can lead to errors in the estimation of *g*_m_ in conditions of changing [CO_2_] (Gilbert et al. 2011). Because of these potential artifacts and errors, it is necessary to confirm previous studies on the response of *g*_m_ to [CO_2_] in angiosperms and ferns through the use of complimentary methods. We chose specifically to look at ferns because of the limited information published on CO_2_ responses and to determine if like stomatal responses to CO_2_, ferns also differed in *g*_m_ responses compared with angiosperms.

The purposes of this study were to determine: (1) the photosynthetic traits of ferns, including *g*_m_ and *g*_s_ at the present [CO_2_] (400 µmol mol^−1^) for comparison against published values for ferns and angiosperms, and (2) the rapid and continuous response of *g*_m_, *g*_s_ and photosynthetic rate of ferns to changing [CO_2_]. For these purposes, we developed a custom-designed gas exchange system using a concurrent measurement of gas exchange and carbon isotope ratio using tunable diode laser absorption spectroscopy (TDLAS), to quantify the rapid, continuous responses in *g*_m_ in fern species in response to changes in [CO_2_] with a time resolution of a few minutes (Tazoe et al. 2009, 2011). O_2_ gas was used at a level of 2 % for gas exchange measurements in order to minimize the effect of photorespiration on carbon isotope measurements. To the best of our knowledge, this is the first study to examine continuous responses in *g*_m_ in fern species in response to changes in [CO_2_]. We also determined leaf anatomical traits using light micrographs and calculated photosynthetic parameters using the light–response curve and *A*/*C*_i_ curve, to compare the photosynthetic traits of ferns with those of angiosperms reported previously.

We selected two fern species from order Polypodiales, *Pteridium aquilinum* and *Thelypteris dentata*. From recent phylogenetic studies, Polypodiales is the most modern order among the seven fern orders in Polypodiopsida (Smith et al. 2006). The estimated divergence time of *Pteridium* (Dennstaedtiaceae family) and *Thelypteris* (Thelypteridiaceae family, Eupolipods II) is ~90 and ~65 million years, respectively (Pryer et al. 2004), when atmospheric CO_2_ levels decreased with time from ~2,000 to ~500 ppm (Bice and Norris 2002). *P. aquilinum* was possibly distributed worldwide in the Oligocene (Der et al. 2009) when the atmospheric CO_2_ levels had decreased (~400 ppm; Zhang et al. 2013). *P.* *aquilinum* and *T. dentata* grow in open sites and show higher photosynthetic rates than those of other ferns that grow in shady sites. High photosynthetic rate assures high accuracy in the estimation of *g*_m_ using the carbon isotope method.

## Materials and methods

### Plants materials

**Fig. 1:**
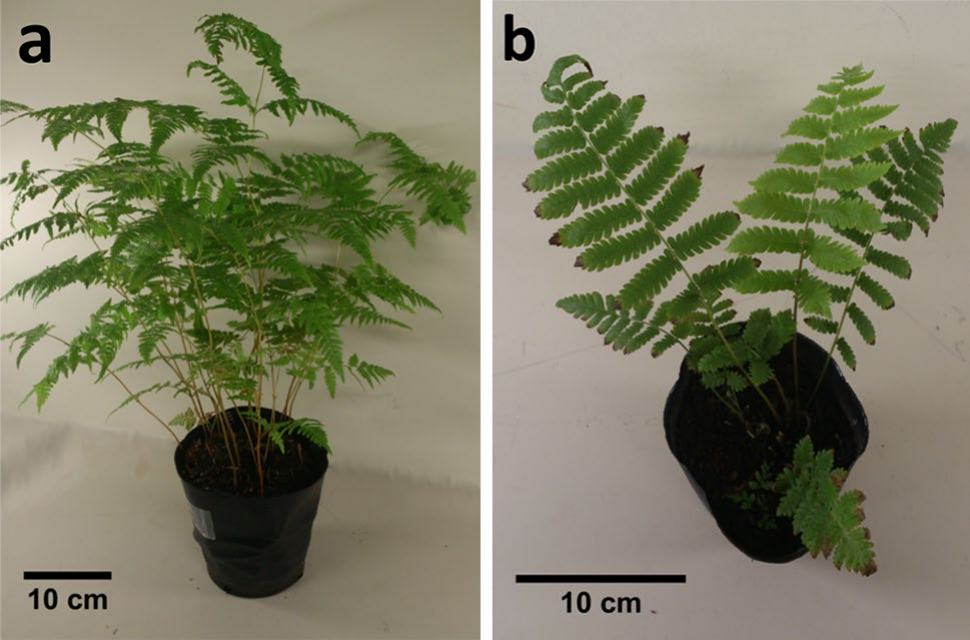
Whole plant images of **a** *Pteridium aquilinum* and **b** *Thelypteris dentata*. 83 × 55 mm (300 × 300 DPI)

*Pteridium aquilinum* (L.) Kuhn (Fig. 1a) and *T. dentata* (Forssk.) E. P. St. John (Fig. 1b) were used. *P. aquilinum* is a deciduous fern that grows in open habitats and is distributed widely in temperate zones in the Northern hemisphere. *T. dentata* is an evergreen fern that grows in open habitats in tropical or subtropical zones, and which has recently expanded into southern coastal areas in Japan (Murakami et al. 2007). Rhizomes of *P. aquilinum* and *T. dentata* were purchased commercially (Takayama Engei, Kyoto, Japan) and collected around the greenhouse at Kyoto Institute of Technology (Ukyo-ku, Kyoto, Japan), respectively. Five rhizomes of each species were planted in 3-liter pots filled with mixed soil (peat moss:humus:sand = 3:3:1 volume ratio) in a 50 % shaded glasshouse. Five plants of both species were used for light–response curve, *A*/*C*_i_ curve, and anatomical analysis. Three or four of the five plants were used for CO_2_ response measurements. Average daytime photosynthetic photon flux density (PPFD) in the glasshouse was 221 ± 7 µmol m^−2^ s^−1^. Plants were watered every 2 days, fertilized with a 1/2,000 solution of Hyponex 6-10-5 (Hyponex Japan, Osaka, Japan) once a month. The gas exchange experiment was carried out in October 2013. Average temperature and relative humidity in the glasshouse from frond emergence to the experiments were 20.7 ± 0.1 °C and 74.5 ± 0.3 %, respectively.

### Estimation of *g*_m_

**Fig. 2:**
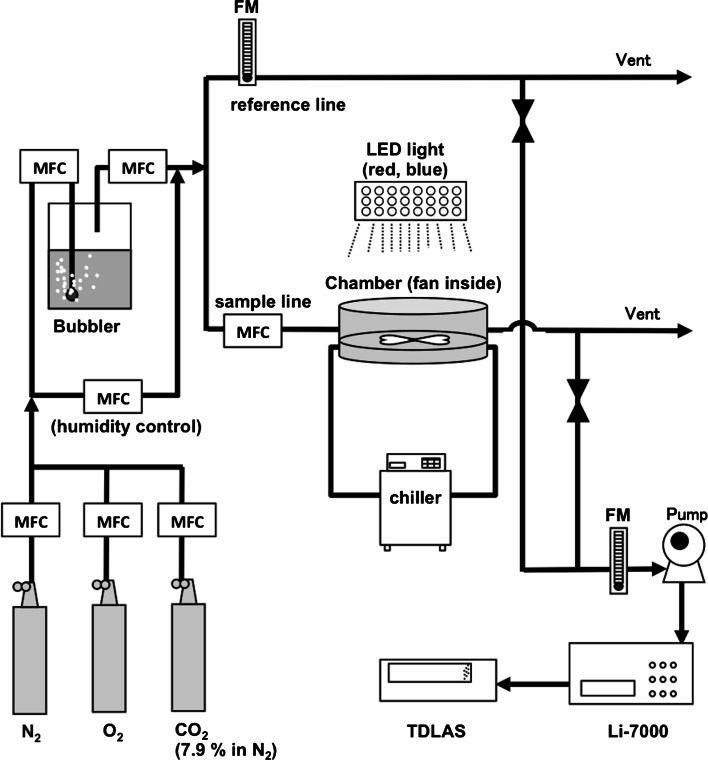
The custom-made gas exchange system connected to a CO_2_ isotope analyzer, a tunable diode laser absorption spectroscope (TDLAS). *MFC* mass flow controller, *FM* flow meter. 120 × 126 mm (299 × 299 DPI)

**Fig. 3:**
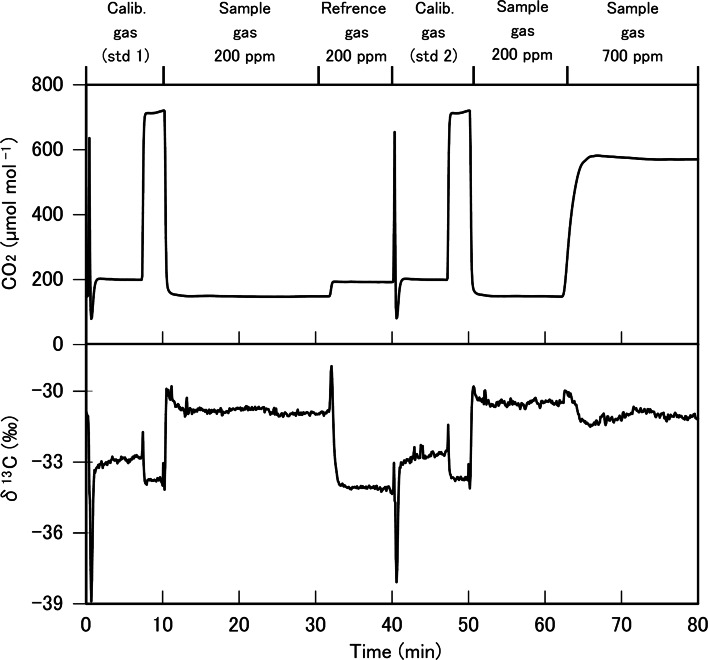
An example of the 80 min cycle of the measurement of carbon isotope ratio using tunable diode laser absorption spectroscopy, where atmospheric CO_2_ concentration [CO_2_] was altered from 200 to 700 μmol mol^−1^. After calibration gas 1 (std 1) was measured, sample and reference gas from the Li-7000 was measured at a reference CO_2_ of 200 μmol mol^−1^, followed by the measurement of calibration gas 2 (std 2). Thereafter, the sample gas was measured at a reference CO_2_ of 200 μmol mol^−1^ again, and then the reference CO_2_ was changed to 700 μmol mol^−1^ and then sample gas was measured. A similar measurement cycle was repeated for the changes in [CO_2_] from 700 to 400 μmol mol^−1^ and from 400 to 200 μmol mol^−1^. 118 × 113 mm (300 × 300 DPI)

Gas exchange and carbon isotope discrimination were measured concurrently using a custom-designed system constructed at the National Institute for Agro-Environmental Sciences (Fig. 2), which was based on previous studies (Nelson et al. 2008; Tuzson et al. 2008; Wada et al. 2011). A custom-made gas exchange system was connected to a CO_2_ isotope analyzer, a tunable diode laser absorption spectroscope (QC-TILDAS-ISO, Aerodyne Research Inc., Billerica, MA, USA) for the sequential measurement of CO_2_ isotopologues. A custom leaf chamber with a diameter of 12 cm with temperature and humidity controlled by a thermo chiller (SMC, HRS018-AF-10) and a bubbler, respectively, was connected to the CO_2_/H_2_O analyzer (Li-7000, LI-COR) for the gas exchange measurements. Leaf area was measured using scanned images of the cut leaves immediately after the measurement, using ImageJ software (http://imagej.nih.gov/ij/). We measured the leaf boundary layer conductance in accordance with the leaf area by obtaining a calibration curve using saturated filter papers with different areas. The fan placed inside the chamber mixed the air in the chamber completely. A thermocouple placed inside the chamber was connected to the gas exchange analyzer in order to record leaf temperature. A red and blue light emitting diode (LED) light source (red: blue 8:1, LEDRB-630DL, Opto Code Corp., Tokyo, Japan) was set onto the chamber, with PPFD of 500 µmol m^−2^ s^−1^ at the leaf surface. The flow rate was set at 500 ml min^−1^, and leaf temperature at 25–28 °C. Vapor pressure deficit (VPD) was set at <1.5 kPa. N_2_ and O_2_ gas were mixed using mass controllers (SEC-E40, HORIBA Ltd., Kyoto, Japan) to generate 2 % O_2_. To determine photosynthetic responses to changes in atmospheric CO_2_ concentration [CO_2_], a fully expanded mature leaf was clamped into the chamber, and the ambient CO_2_ concentration (*C*_a_) was kept at 400 µmol mol^−1^ for 40–60 min. After that, *C*_a_ was first reduced to 200 µmol mol^−1^ for 80 min, and then increased to 700 μmol mol^−1^ for 80 min. Finally, *C*_a_ was decreased to 400 µmol mol^−1^. We varied *C*_a_ from 200 to 700 μmol mol^−1^ because leaf photosynthesis of ferns was CO_2_-limited in this range of *C*_a_ in *A*/*C*_i_ curve analysis. Measurements were performed at 30 s intervals, calibrated every 30 min using two standard gas cylinders, 200 and 700 μmol mol^−1^, during the measurement (Fig. 3). Stability of TDLAS was tested using standard CO_2_ gas at a CO_2_ concentration of 400 μmol mol^−1^ for 2 h before and after the measurements. Analysis with Allan variance showed that deviation of δ^13^C for 30 min was <0.03 %. The δ^13^C of the gas was stabilized completely within 10 min. Observed carbon isotope discrimination during photosynthesis ($\delta_{o}$) was calculated using the following equation (Evans et al. 1986),

1 $$\delta_{o} = \frac{{ 1 0 0 0 \xi \left( {\delta {}_{{}}^{ 1 3} C_{a} {-}\delta {}_{{}}^{ 1 3} C_{\text{ref}} } \right)}}{{ 1 0 0 0\,{ + }\,\delta {}_{{}}^{ 1 3} C_{a} {-}\xi \left( {\delta {}_{{}}^{ 1 3} C_{a} {-}\delta {}_{{}}^{ 1 3} C_{\text{ref}} } \right)}}$$

where *δ*^13^*C*_*a*_ and *δ*^13^*C*_ref_ are the carbon isotope composition in the leaf chamber and in reference air. ξ = *C*_ref_/(*C*_ref_ – *C*_a_), where *C*_a_ and *C*_ref_ are the CO_2_ concentration in the leaf chamber and in reference air. ξ was kept at <9 during measurements in order to ensure high precision and accuracy for *g*_m_ estimation (Pons et al. 2009). Mesophyll conductance was calculated using the equations reported by Evans and von Caemmerer (2013) assuming no photorespiration:

2 $$g_{m} = \frac{{ 1 { + }t}}{{ 1 {-}t}}\left( {b{-}a_{i} {-}\frac{{eR_{d} }}{{\left( {A + R_{d} } \right)}}} \right)\frac{A}{{C_{a} }}/\left( {\delta_{i} {-}\delta_{o} {-}\delta_{e} } \right)$$

$t =\left( { 1 { + }a'} \right)E/2g_{\text{ac}}^{t}$, where *a*′ is a combined fractionation factor through the boundary layer and stomata,

3 $$a ' =\frac{{a_{b} (C_{a} {-}C_{s} ) { + }a(C_{s} {-}C_{i} )}}{{(C_{a} {-}C_{i} )}}$$

where *a*_b_ (2.9 ‰) and *a* (4.4 ‰) are the fractionation through CO_2_ diffusion in the boundary layer and air, respectively (Evans et al. 1986). *C*_s_ and *C*_i_ are the CO_2_ concentration at the leaf surface and in the leaf intercellular air space, respectively. *E* is the transpiration rate, and *g*_ac_^t^ is total conductance to CO_2_ diffusion. *b* (30 ‰) is the fractionation associated with Rubisco carboxylation (Roeske and O’Leary 1984), *a*_i_ (1.8 ‰) is the fractionation factor for dissolution and diffusion through water (O’Leary 1981), and *R*_d_ is day respiration. The parameter *e*, which is associated with day respiration, was calculated as *e* = *δ*^13^*C*_tank_ − *δ*^13^*C*_atmosphere_, assuming no fractionation by day respiration (Evans and von Caemmerer 2013; Tazoe et al. 2009). *δ*^13^*C*_tank_ was from −34 to −36 ‰, and *δ*^13^*C*_atmosphere_ was assumed to be −8 ‰. $\delta_{i}$ is fractionation when *C*_i_ = *C*_c_ without respiratory fractionation:

4 $$\delta_{i} = \frac{1}{{ ( 1 {-}t)}}a ' { + }\frac{1}{{ ( 1 {-}t)}}\left( {\left( { 1 { + }t} \right)b{-}a'} \right)\frac{{C_{i} }}{{C_{a} }}$$

$\delta_{e}$ is fractionation with respiration, which was calculated as:

5 $$\delta_{e} = \frac{{ 1 { + }t}}{{ 1 {-}t}}\left( {\frac{{eR_{d} }}{{\left( {A + R_{d} } \right)C_{a} }}\left( {C_{i} {-}\varGamma^{*} } \right)} \right)$$

Γ^*^ is the CO_2_ compensation point in the absence of *R*_d_, which was estimated following the procedure reported by Laisk et al. (1984). We used C*, the apparent CO_2_ compensation point provided by Laisk et al. (1984), as a proxy of Γ^*^ (Douthe et al. 2011). C^*^ was 65.1 ± 3.9 and 65.1 ± 4.4 μmol mol^−1^ in *P. aquilinum* and *T. dentata,* respectively, and *R*_d_ was 3.8 ± 0.2 and 3.4 ± 0.2 μmol m^−2^ s^−1^, respectively.

### Estimation of photosynthetic parameters

Leaf photosynthetic parameters were estimated from the light–response curve model (Ogren and Evans 1993) and the *A*/*C*_i_ curve fitting method (*A*/*C*_i_ Curve Fitting 10.0.xls, http://landflux.org/Tools.php, Ethier and Livingston 2004; Ethier et al. 2006) using a photosynthesis system (Li-6400, LI-COR). Leaf temperature and vapor pressure deficit (VPD) were set at 25 °C and 1.5 kPa, respectively. *C*_a_ was 400 μmol mol^−1^ for the light–response curve analysis. PPFD was decreased stepwise from 500 to 0 μmol m^−2^ s^−1^. Thereafter, PPFD was returned to 400 μmol m^−2^ s^−1^ and then increased stepwise to 1,500 μmol m^−2^ s^−1^. Light-saturated photosynthesis rate (*A*_sat_), curvature factor, quantum use efficiency, dark respiration rate, light compensation point, and *g*_s_ were obtained from light–response curves. For the *A*/*C*_i_ curve analysis, PPFD was set at 1,000 and 500 μmol m^−2^ s^−1^ for *P. aquilinum* and *T. dentata*, respectively, because our previous experiment obtained saturated PPFD of 1,000 and 500 μmol m^−2^ s^−1^ for *P. aquilinum* and *T. dentata*, respectively. *C*_a_ was decreased stepwise from 400 to 50 μmol mol^−1^, then returned to 400 μmol mol^−1^, and increased stepwise to 2,000 μmol mol^−1^. Maximum carboxylation rate (*V*_cmax_) and electron transport rate (*J*) were calculated from the *A*/*C*_i_ curve, assuming constant *g*_m_ during the changes in *C*_i_.

### Analysis of leaf morphological traits

Leaf mass per area was calculated as leaf dry weight divided by leaf area. Leaf mesophyll anatomy was determined using light and transmission electron micrographs. Leaf sections of 2 × 3 mm were fixed in 5 % glutaraldehyde and 1 % osmium tetroxide, and were embedded in Spurr’s resin (Low Viscosity Resin kit, TAAB, Aldermaston, UK). Transverse Sects. (800 nm thick) were stained with 1 % toluidine blue solution. Anatomical characteristics were determined from digitized images of micrographs taken at ×400 magnification (BX51-33, OLYMPUS, Tokyo, Japan). The surface area of mesophyll cells and chloroplasts exposed to intercellular air spaces per unit leaf area (*S*_mes_ and *S*_c_) were estimated for transverse sections as described by Hanba et al. (2002). Transverse Sects. (70 nm thick) were stained with 2 % uranyl acetate and Reynold’s lead citrate. Thickness of cell walls covered with chloroplasts (cell wall thickness), and chloroplast thickness and width were measured from ×6,000 and ×2,500 magnification images, respectively, from micrographs taken by a transmission electron microscope (JEM-1220, JOEL, Tokyo, Japan), analyzed using ImageJ software (http://imagej.nih.gov/ij/).

### Statistical analysis

Differences in mean values between species were tested using an unpaired *t* test to analyze photosynthetic parameters and leaf morphological traits. The effect of [CO_2_] on leaf gas exchange was analyzed using an unpaired *t* test. These statistical analyses were conducted using EZR version 1.24 (Kanda 2013; http://www.jichi.ac.jp/saitama-sct/SaitamaHP.files/statmedEN.html).

## Results

**Fig. 4:**
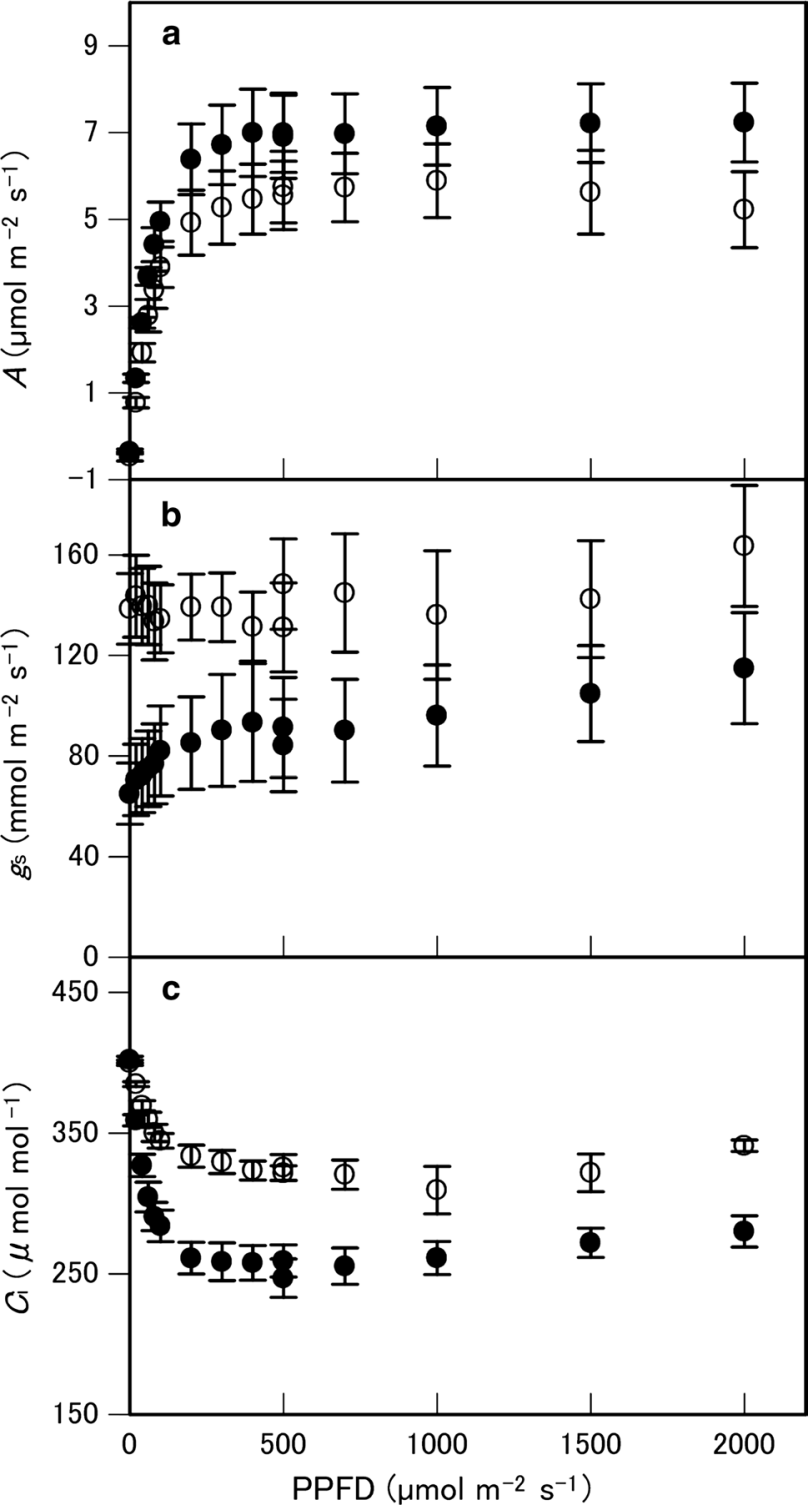
Changes in **a** leaf photosynthesis rate (*A*), **b** stomatal conductance (*g*_s_), and intercellular CO_2_ concentration (*C*_i_), **c** against photosynthetic photon flux density (PPFD) in *Pteridium aquilinum* (*filled circles*) and *Thelypteris dentata* (*open circles*) at 400 μmol mol^−1^ of ambient atmospheric CO_2_. Data points are means with bars for standard errors (*n* = 5). 81 × 156 mm (300 × 300 DPI)

**Fig. 5:**
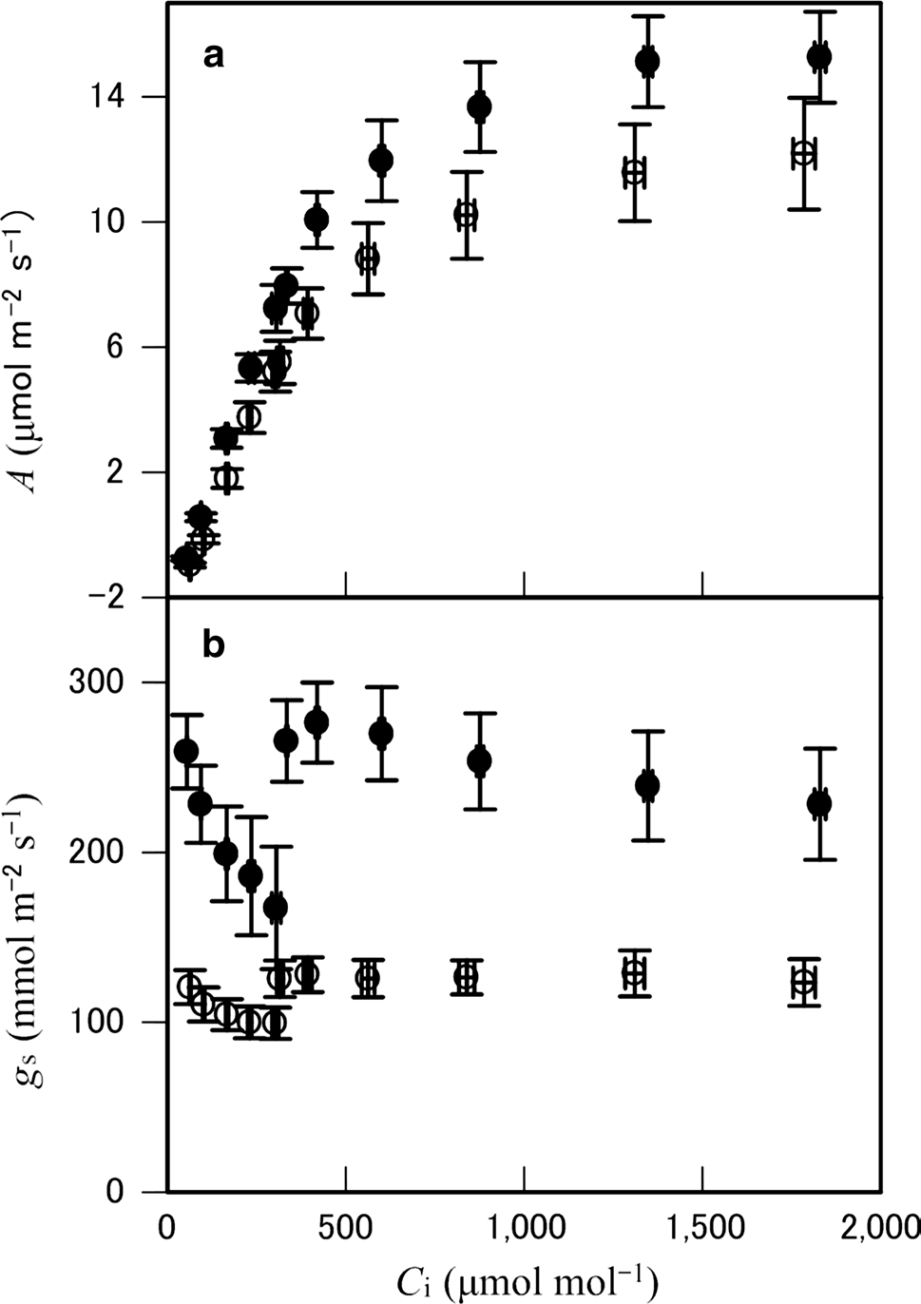
Response of **a** photosynthesis rate (*A*) and **b** stomatal conductance (*g*_s_) to intercellular CO_2_ concentration (*C*_i_) in *Pteridium aquilinum* (*filled circles*) and *Thelypteris dentata* (*open circles*). Ambient CO_2_ concentration was first decreased from 400 to 50 μmol mol^−1^, then returned to 400 μmol mol^−1^ and increased to 2000 μmol mol^−1^. Data points are means with bars for standard errors (*n* = 5)

Photosynthesis rate (*A*) was light saturated at a PPFD of 500 μmol m^−2^ s^−1^ for both *P. aquilinum* and *T.* *dentata* (Fig. 4a). For *T. dentata*, *A* tended to decrease when PPFD exceeded 1,000 μmol m^−2^ s^−1^. There were no significant differences in *A*_sat_, curvature factor, quantum use efficiency, respiration rate, or light compensation point between the two species (Table 1). When PPFD decreased from 500 to 0 μmol m^−2^ s^−1^, *g*_s_ in *P. aquilinum* tended to decrease but that of *T. dentata* was almost constant (Fig. 4b), with a significant increase in intercellular CO_2_ concentration (*C*_i_) in both species (*P* < 0.05; Fig. 4c). The *g*_s_ of *P. aquilinum* was compared with that of *T. dentata* and showed no significant difference at a PPFD of 2,000 μmol m^−2^ s^−1^. *V*_max_ and *J* calculated using *A*/*C*_i_ curves were also not significantly different between the two species (Fig. 5a; Table 1). When *C*_i_ was decreased from 300 to 50 μmol mol^−1^, the *g*_s_ of both species significantly increased (*P* < 0.05; Fig. 5b). With *C*_i_ of 400 μmol mol^−1^ to 1,800 μmol mol^−1^, *g*_s_ values remained almost constant in both species.

**Table 1:** Photosynthetic parameters obtained from the light-response and *A*/*C*_i_ curves of the ferns *Pteridium aquilinum* and *Thelypteris dentata*

Parameters	*P. aquilinum*	*T. dentata*	*P*
*A*_sat_ (μmol m^−2^ s^−1^)	7.54 ± 0.93	6.24 ± 0.90	n.s.
Curvature factor	0.87 ± 0.02	0.83 ± 0.06	n.s.
Quantum use efficiency (μmol CO_2_ μmol photon^−1^)	0.074 ± 0.003	0.061 ± 0.006	n.s.
Dark respiration rate (μmol m^−2^ s^−1^)	0.35 ± 0.06	0.46 ± 0.10	n.s.
Light compensation point (μmol m^−2^ s^−1^)	3.98 ± 0.66	7.47 ± 1.89	n.s.
*g*_s_ (mmol m^−2^ s^−1^) at PPFD of 2,000 μmol m^−2^ s^−1^	115.0 ± 22.1	163.6 ± 24.1	n.s.
*V*_max_ (μmol m^−2^ s^−1^)	40.4 ± 3.7	35.6 ± 5.4	n.s.
*J* (μmol m^−2^ s^−1^)	72.0 ± 6.8	58.8 ± 8.1	n.s.

*V*_*max*_ and *J* were obtained from *A*/*C*_i_ curves in accordance with Ethier and Livingston (2004). Values are mean ± SE from five different plants (*n* = 5). Statistical analysis was done using *t* test

*A*_*sat*_ curvature factor, quantum use efficiency, dark respiration rate and light compensation point were calculated from light–response curves in accordance with Ogren and Evans (1993), *n.s.* not significant

**Fig. 6:**
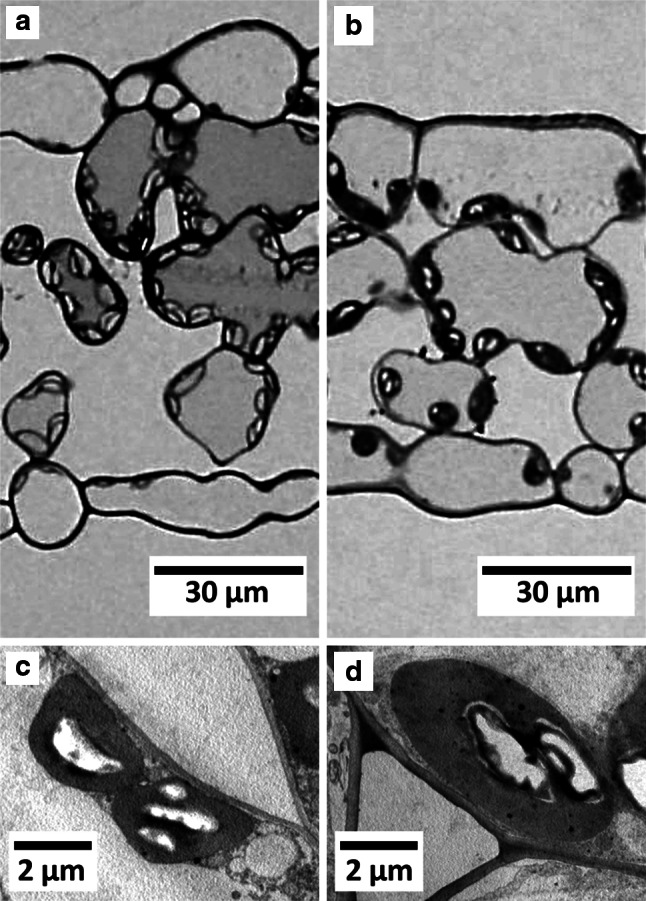
Light micrograph of transverse leaf sections of **a** *Pteridium aquilinum* and **b** *Thelypteris dentata* at 400× magnification. Transmission electron microscope images of **c** *Pteridium aquilinum* and **d** *Thelypteris dentata* at 6,000× magnification. 83 × 117 mm (300 × 300 DPI)

Transverse sections of the fronds of *P. aquilinum* (Fig. 6a, c) and *T. dentata* (Fig. 6b, d) showed that both ferns had loosely packed mesophyll cells and lacked distinct palisade tissue. Both ferns also had chloroplasts in the upper and lower epidermal cells. The width and thickness of chloroplasts in *T. dentata* were significantly larger than those of *P. aquilinum* (Table 2). The chloroplasts had large spaces between them and, as a result, 59 and 44 % of *S*_mes_ were not covered with chloroplasts for *P. aquilinum and T.* *dentata*, respectively. The *S*_mes_ and internal air spaces of *P. aquilinum* were significantly larger than those of *T. dentata* (Table 2). Other traits including *S*_c_, LMA, leaf thickness, cell wall thickness, and chloroplast width/thickness were not significantly different between the two species.

**Table 2:** Leaf morphological traits of the ferns *Pteridium aquilinum* and *Thelypteris dentata*

Parameters	*P. aquilinum*	*T. dentata*	*P*
*S*_c_ (m^2^ m^−2^)	3.9 ± 0.6	5.0 ± 0.4	n.s.
*S*_mes_ (m^2^ m^−2^)	11.0 ± 0.6	9.0 ± 0.3	0.01
Leaf mass per area (g m^−2^)	31.7 ± 3.1	39.7 ± 4.0	n.s.
Leaf thickness (μm)	163.4 ± 9.1	149.9 ± 9.3	n.s.
Internal air space (%)	39.6 ± 5.5	20.8 ± 2.8	0.02
Cell wall thickness (μm)	0.23 ± 0.02	0.30 ± 0.03	n.s.
Chloroplast width (μm)	4.51 ± 0.23	6.65 ± 0.26	<0.01
Chloroplast thickness (μm)	2.43 ± 0.21	3.97 ± 0.07	<0.01
Chloroplast width/thickness	1.94 ± 0.15	1.68 ± 0.04	n.s.

Values are mean ± SE from five different plants (*n* = 5). Statistical analysis was done using *t* test

*n.s.* not significant

**Fig. 7:**
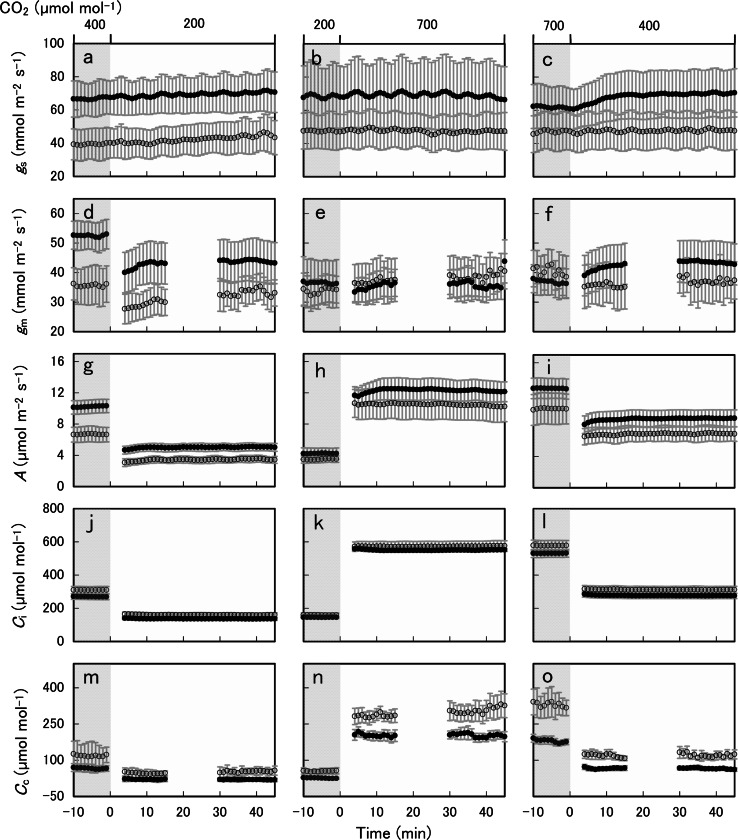
Changes in stomatal conductance (*g*_s_), mesophyll conductance (*g*_m_), photosynthesis rate (*A*), intercellular CO_2_ concentration (*C*_i_) and chloroplast CO_2_ concentration (*C*_c_) in response to atmospheric CO_2_ concentration [CO_2_] for *Pteridium aquilinum* (*filled circles*) and *Thelypteris dentata* (*open circles*). [CO_2_] was first decreased from 400 to 200 μmol mol^−1^ (*left panels*), then increased from 200 to 700 μmol mol^−1^ (*middle panels*), and finally decreased from 700 to 400 μmol mol^−1^ (*right panels*). Data points were averaged for two data (1 min) from 3 or 4 different plants with *bars* for standard errors (*n* = 3 or 4). 167 × 196 mm (300 × 300 DPI)

When atmospheric CO_2_ concentration [CO_2_] was decreased from 400 to 200 μmol mol^−1^, *g*_s_ of both ferns increased slightly with time (Fig. 7a), with a 3.7 mmol m^−2^ s^−1^ increase in *P. aquilinum* and a 5.4 mmol m^−2^ s^−1^ increase in *T. dentata* on average (Table 3). In contrast, *g*_m_ of both ferns decreased rapidly (Fig. 7d), with a 8.6 mmol m^−2^ s^−1^ decrease in *P. aquilinum* and a 2.1 mmol m^−2^ s^−1^ decrease in *T. dentata* (Table 3). *A* and *C*_i_ of *P. aquilinum* and *T. dentata* also showed rapid decreases of 5.1 and 3.2, 134.2, and 150.8 μmol m^−2^ s^−1^, respectively (Fig. 7 g, j). The decrease in *C*_c_ was 47 and 65.1 μmol mol^−1^ in *P. aquilinum* and *T. dentata*, respectively (Fig. 7 m).

**Table 3:** The response of stomatal conductance (*g*_s_), mesophyll conductance (*g*_m_), photosynthesis rate (*A*), intercellular CO_2_ concentration (*C*_i_), and chloroplast CO_2_ concentration (*C*_c_) to [CO_2_]

CO_2_ (μmol mol^−1^)		400	200		200	700		700	400	
*g*_s_ (mmol m^−2^ s^−1^)	*P. aquilinum*	66.8 ± 0.1	70.5 ± 0.2	*P* < 0.01	68.4 ± 0.2	68.2 ± 0.2	n.s.	61.8 ± 0.1	70.1 ± 0.1	*P* < 0.01
	*T. dentata*	39.3 ± 0.1	44.7 ± 0.2	*P* < 0.01	47.4 ± 0.1	47.2 ± 0.1	n.s.	47.0 ± 0.2	47.9 ± 0.2	*P* < 0.01
*g*_m_ (mmol m^−2^ s^−1^)	*P. aquilinum*	52.5 ± 0.1	43.9 ± 0.1	*P* < 0.01	36.5 ± 0.1	35.4 ± 0.2	*P* < 0.01	36.9 ± 0.1	43.4 ± 0.1	*P* < 0.01
	*T. dentata*	35.6 ± 0.1	33.5 ± 0.3	*P* < 0.01	33.8 ± 0.3	38.7 ± 0.3	*P* < 0.01	40.5 ± 0.4	33.9 ± 0.1	*P* < 0.01
*A* (μmol m^−2^ s^−1^)	*P. aquilinum*	10.2 ± 0.0	5.1 ± 0.0	*P* < 0.01	4.2 ± 0.0	12.2 ± 0.0	*P* < 0.01	12.7 ± 0.0	8.7 ± 0.0	*P* < 0.01
	*T. dentata*	6.7 ± 0.0	3.5 ± 0.0	*P* < 0.01	3.5 ± 0.0	10.3 ± 0.0	*P* < 0.01	10.1 ± 0.0	6.9 ± 0.0	*P* < 0.01
*C*_i_ (μmol mol^−1^)	*P. aquilinum*	269.6 ± 0.2	135.4 ± 0.1	*P* < 0.01	144.8 ± 0.1	550.3 ± 0.3	*P* < 0.01	530.4 ± 0.1	275.0 ± 0.1	*P* < 0.01
	*T. dentata*	309.3 ± 0.1	158.5 ± 0.2	*P* < 0.01	158.9 ± 0.2	573.8 ± 0.3	*P* < 0.01	577.0 ± 0.2	310.4 ± 0.1	*P* < 0.01
*C*_c_ (μmol mol^−1^)	*P. aquilinum*	66.0 ± 0.6	19.0 ± 0.1	*P* < 0.01	25.7 ± 0.3	200.7 ± 1.7	*P* < 0.01	179.6 ± 1.4	64.5 ± 0.5	*P* < 0.01
	*T. dentata*	118.1 ± 0.9	53.0 ± 0.8	*P* < 0.01	54.2 ± 0.9	308.7 ± 3.3	*P* < 0.01	329.6 ± 2.3	116.4 ± 1.6	*P* < 0.01

Values are mean ± SE from three or four different plants, averaged over −10 to 0 min and 35–45 min. Time “0” means that [CO_2_] was changed at that time. Statistical analyses between before and after changing [CO_2_] were done using the Student’s *t* test. The data at 30 s intervals was used for the analysis

When [CO_2_] was increased from 200 to 700 μmol mol^−1^, *g*_s_ did not change in either species (Fig. 7b; Table 3). *g*_m_ of *P. aquilinum* decreased by 1.1 mmol m^−2^ s^−1^, whereas it increased by 4.9 mmol m^−2^ s^−1^ in *T.* *dentata* (Table 3; Fig. 7e). *A* and *C*_i_ increased rapidly, by 8 and 6.8 μmol m^−2^ s^−1^ and 405.5 and 414.9 μmol mol^−1^ for *P. aquilinum* and *T. dentata*, respectively (Fig. 7 h, k), and the respective increases in *C*_c_ were 175 and 254.5 μmol mol^−1^ (Fig. 7n).

When [CO_2_] was decreased from 700 to 400 μmol mol^−1^, *g*_s_ of both species increased slightly with time (Fig. 7c), with a 8.3 mmol m^−2^ s^−1^ increase in *P. aquilinum* and a 0.9 mmol m^−2^ s^−1^ increase in *T.* *dentata* on average (Table 3). The *g*_m_ of *P. aquilinum* increased by 6.5 mmol m^−2^ s^−1^, whereas that of *T.* *dentata* decreased by 6.6 mmol m^−2^ s^−1^ on average (Table 3; Fig. 7f). *A* and *C*_i_ decreased rapidly, by 4.0 and 3.2 and 255.4 and 266.6 μmol mol^−1^ for *P. aquilinum* and *T.* *dentata*, respectively (Fig. 7i, l), and the respective decreases in *C*_c_ were 115.1 and 213.2 μmol mol^−1^ (Fig. 7o).

## Discussion

### Steady-state leaf photosynthetic traits and morphology in ferns

There was no significant difference in photosynthesis traits between *P. aquilinum* and *T. dentata* obtained from the analysis of light–response curves and *A*/*C*_i_ curves (Table 1). *P. aquilinum* and *T. dentata* are Polypodiales, which is the most modern among the fern orders (Smith et al. 2006). Photosynthesis traits were compared with those reported in previous studies of fern species in Polypodiales. *A*_sat_, dark respiration rate, light compensation point, *V*_max_, and *J* were within the range of those reported in previous studies, although the growth conditions differed (Carriquí et al. 2015; Gago et al. 2013; Sessa and Givnish 2014). At present, there is little evidence that photosynthetic traits are different between phylogenetically distant fern groups; two basal ferns *Equisetum telmateia* (Equisetales) and *O. regalis* (Osmundales) had photosynthetic traits within the range of those in Polypodiales (Carriquí et al. 2015; Gago et al. 2013). However, photosynthesis data for basal ferns are so scarce that further systematic studies are needed for phylogenetic consideration. The present result confirmed that the photosynthetic capacity of ferns is generally lower than angiosperms; ferns had lower *A*_sat_, dark respiration rate, *g*_s_, *V*_max_, and *J* than angiosperms (Carriquí et al. 2015). The values reported in this study are in the range of those reported in Carriquí et al. (2015) for fern species.

Although photosynthesis traits are similar between *P. aquilinum* and *T. dentata*, internal air spaces and *S*_mes_ were larger in *P. aquilinum* than in *T. dentata*, because of its loosely packed mesophyll cells. The smaller size of chloroplasts in *P. aquilinum* (Table 2) offset the effect of higher *S*_mes_, which involves similar *S*_c_ between *P. aquilinum* and *T. dentata.* This similar *S*_c_ may relate to the similar photosynthetic traits between species in the present study. Compared with angiosperms, where the lowest *S*_c_ so far reported was 5.0 m^2^ m^−2^ in a tree species *Acer rufinerve* grown in shade (Hanba et al. 2001), the *S*_c_ measurements of fern species here were among the lowest values, with an *S*_c_ of 3.9 ± 0.6 m^2^ m^−2^ in *P.* *aquilinum* and 5.0 ± 0.4 m^2^ m^−2^ in *T. dentata*. Terashima et al. (2006) reported that in seed plants, the average *S*_c_ was 15.01 m^2^ m^−2^ in annuals, 12.05 m^2^ m^−2^ in deciduous trees, and 14.45 m^2^ m^−2^ in evergreen trees. Carriquí et al. (2015) reported that the average *S*_c_ for seven angiosperms and ferns was 10.3 and 7.6 m^2^ m^−2^, respectively. These previous studies, together with our results, indicate that the *S*_c_ of ferns is lower than the *S*_c_ of most angiosperms. Small *S*_c_ measurements in fern species are related to small mesophyll thickness with large intercellular airspaces, and may also be partly affected by the size of chloroplasts (Table 2). *S*_c_ is one of the most significant factors affecting *g*_m_, where high *S*_c_ allows plants to increase diffusion of CO_2_ into chloroplasts. Angiosperms had much lower atmospheric CO_2_ levels than ferns at their emergence period, and this may have been crucial for angiosperms to increase diffusional surface for CO_2_ to achieve high photosynthetic rates.

Cell wall thickness was 0.23 ± 0.02 μm in *P. aquilinum* (Table 2), which is similar to the cell wall thickness of 0.194 μm in *P. aquilinum* reported by Carriquí et al. (2015). The cell wall thickness of *P.* *aquilinum* and *T. dentata* obtained here (0.23 ± 0.02 μm and 0.30 ± 0.03 μm) are in the range of typical values for deciduous trees (0.2–0.3 μm; Terashima et al. 2006). Carriquí et al. (2015) reported higher averaged values of cell wall thickness in ferns (0.359 μm) than those in angiosperms (0.251 μm) and suggested an evolutionary trend towards reduced thickness from ferns to angiosperms. More evidence is needed to substantiate this evolutionary trend, considering the uncertainties in estimation of cell wall thickness using electron micrographs and large variability among the same phylogenic group (from 0.194 to 0.687 μm in ferns, Carriquí et al. 2015). LMAs of *P. aquilinum* and *T. dentata* were much smaller than those in tree species (Hanba et al. 1999; Tomás et al. 2013), which may also be partly affected by the low mesophyll thickness in ferns.

### Steady-state *g*_m_ of fern species compared with angiosperms at the present [CO_2_]

One of the goals of our study was to determine steady-state *g*_m_ values of ferns and compare them with the published values for angiosperms at the present [CO_2_] (400 μmol mol^−1^). For the estimation of *g*_m_, carbon isotope, chlorophyll fluorescence, and *A*/*C*_i_ curve-fitting methods have all been used previously (Pons et al. 2009). There are only three studies that reported the *g*_m_ of ferns. Gago et al. (2013) showed that the *g*_m_ of *O. regalis*, *B. gibbum* and *N. exaltata* were between 30 and 73 mmol m^−2^ s^−1^ using a chlorophyll fluorescence method, and between 30 and 112 mmol m^−2^ s^−1^ using an *A*/*C*_i_ curve-fitting method. Volkova et al. (2009) showed that *g*_m_ of *Dicksonia antarctica* grown in the shade and at high irradiance was 115 ± 35 and 155 ± 64 mmol m^−2^ s^−1^, respectively, estimated from *A*/*C*_i_ curve fitting. Carriquí et al. (2015) reported that *g*_m_ varies from 26 to 253 mmol m^−2^ s^−1^ for seven fern species using a chlorophyll fluorescence method. We used a different method from previous studies reporting the *g*_m_ of ferns (Carriquí et al. 2015; Gago et al. 2013; Volkova et al. 2009). The *g*_m_ values in the present study (35.6 to 52.5 mmol m^−2^ s^−1^) were among the lowest values reported from these three previous studies. Furthermore, the *g*_m_ values of *P. aquilinum* and *T. dentata* in the present study were lower than those of typical seed plants, including angiosperms and gymnosperms (Flexas et al. 2008, 2012).

The cause of the low *g*_m_ in fern species remains to be clarified, but some anatomical traits have been suggested to play a role (Gago et al. 2013; Carriquí et al. 2015). As previously described, *S*_c_ values in *P.* *aquilinum* and *T. dentata* (3.9 ± 0.6 and 5.0 ± 0.4 m^2^ m^−2^) were lower than those of most seed plants including annuals (15.0 m^2^ m^−2^), deciduous broadleaved trees (12.1 m^2^ m^−2^), and evergreen trees (14.5 m^2^ m^−2^) (Terashima et al. 2006). When anatomical traits in the present study were compared with those of an angiosperm, *Lysimachia minoricensis* that had similar LMA (31.4 g m^−2^) to our study (31.7 and 39.7 g m^-2^), the cell wall thickness was similar (0.213 μm and 0.21**–**0.28 μm), but *S*_c_ was much smaller in the present study (3.9 and 5.0 m^2^ m^−2^) than *L. minoricensis* (8.9 m^2^ m^−2^; Carriquí et al. 2015). Therefore, the low *g*_m_ of fern species may be at least partly affected by low *S*_c_; a significant positive correlation was obtained between *g*_m_ and *S*_c_, for angiosperms (Terashima et al. 2006, 2011). The concentration and activities of membrane proteins that transport CO_2_ into mesophyll cells, such as aquaporins (Hanba et al. 2004; Kawase et al. 2013; Terashima and Ono 2002), could also account for the low *g*_m_ of ferns.

### Dynamic response of *g*_s_ and *g*_m_ in response to changes in [CO_2_]

The slight increase in *g*_s_ (<10.4 %) followed a decrease in *C*_a_ from 400 to 200 μmol mol^−1^ (Table 3; Fig. 7). This was consistent with Brodribb et al. (2009), who reported that six ferns/lycopods showed small increases in *g*_s_ when *C*_a_ decreased from 380 to 100 μmol mol^−1^. In a study on *B. gibbum* and *O. regalis,* Gago et al. (2013) reported a slight decrease in *g*_s_ when *C*_a_ decreased from 400 to 50 μmol.mol^−1^, and a similar result was obtained in our study (Fig. 5b). No change was observed in *g*_s_ after an increase in *C*_a_ (from 200 to 700 μmol mol^−1^). This result supports Brodribb et al. (2009), who reported no significant changes in *g*_s_ when *C*_a_ was increased from 380 to 600 μmol mol^−1^ for three Polypodiales ferns. The insensitivity of stomata to the increase in *C*_a_ contrasts with the response seen in angiosperms, which showed a significant decrease (Brodribb et al. 2009). Although the physiological control of stomatal response to [CO_2_] in angiosperms remains poorly understood, Brodribb et al. (2009) hypothesized that a signaling pathway between mesophyll and guard cells in response to [CO_2_] may be present in angiosperms but not in ferns. However, the slight increase in *g*_s_ in ferns following decreased *C*_a_, is similar (but less distinct) to the response in angiosperms, suggesting that the mechanisms involved in CO_2_ sensing in angiosperms may be partly functional in ferns.

In contrast to the increase in *g*_s_, the *g*_m_ of the two fern species in the present study decreased quickly when *C*_a_ was decreased from 400 to 200 μmol mol^−1^ (Fig. 7d). Decreased *g*_m_ at low *C*_i_ (<200 μmol mol^−1^) has been reported for some angiosperms (Flexas et al. 2007; Vrábl et al. 2009) and for three ferns (Gago et al. 2013) using chlorophyll fluorescence or isotope methods. However, previous studies pointed out that the decrease in *g*_m_ at low *C*_i_ might be because of artifacts caused by respiration and photorespiration, when measurements were performed at atmospheric O_2_ level (20 %, Gago et al. 2013; Tholen et al. 2012). As far as we know, three previous studies have been conducted to estimate the *g*_m_ response to [CO_2_] using 2 % or 1 % O_2_ to minimize the effect of photorespiration (Mizokami et al. 2015; Tazoe et al. 2009, 2011). Tazoe et al. (2009, 2011) reported that in some angiosperms, *g*_m_ did not decrease significantly in response to an instantaneous reduction of *C*_i_. Mizokami et al. (2015) reported that in *Nicotiana plumbaginifolia*, both *g*_m_ and *g*_s_ decreased with *C*_i_ on the application of abscisic acid (ABA), but they increased in the absence of ABA, which suggested that *g*_s_ has an effect on *g*_m_ and its response to [CO_2_] (Tazoe and Santrucek 2015). In our study, however, the rapid decrease in *g*_m_ after decreasing *C*_a_ is in contrast to the gradual and slight increase in *g*_s_ for two fern species (Fig. 7a, d). The effect of photorespiration was minimized, because we measured gas exchange at 2 % O_2_. The present result suggested that the decrease in *g*_m_ at 200 μmol mol^−1^ of *C*_a_ in the two ferns may be independent of *g*_s_.

The *g*_m_ did not decrease from 200 to 700 μmol mol^−1^ with [CO_2_] for *T. dentata* and *P. aquilinum* (Fig. 7e; Table 3), which is in contrast to the results that reported significant decreases in *g*_m_ from 200 to 1,000 μmol mol^−1^ of [CO_2_] for three angiosperms (Tazoe et al. 2011). Although a trend where *g*_m_ declines at high [CO_2_] is frequently reported in angiosperms (Bunce 2010; Douthe et al. 2011; Flexas et al. 2007; Mizokami et al. 2015; Vrábl et al. 2009) and also in ferns (Gago et al. 2013), the dependence of *g*_m_ on CO_2_ varied between studies and species. *g*_m_ was highest at <200 μmol mol^−1^ of *C*_i_ in *O. regalis*, whereas *N.* *exaltata* showed highest *g*_m_ at *C*_i_ of 400 μmol mol^−1^ (Gago et al. 2013). These results suggest that plant species have “optimum” *g*_m_ at different CO_2_ levels, which might reflect atmospheric CO_2_ levels during their evolution. *P. aquilinum* showed its highest *g*_m_ at [CO_2_] of 400 μmol mol^−1^ (Table 3), with this species distributed worldwide in the Oligocene (Der et al. 2009) when the CO_2_ level had decreased near to the present level (~400 ppm; Zhang et al. 2013). *T. dentata* showed its highest *g*_m_ at [CO_2_] of 700 μmol mol^−1^ (Table 3), and the estimated divergence time of *T. dentata* is ~65 million years (Pryer et al. 2004) when CO_2_ levels were high (~1,000 ppm; Bice and Norris 2002).

The physiological mechanisms for *g*_m_ responses to changes in [CO_2_] have not been identified for angiosperms or ferns. Mizokami et al. (2015) suggested that the decrease in *g*_m_ in response to the increase in [CO_2_] may be mediated by inactivation of the plasma membrane intrinsic proteins via carbonic anhydrase activity. However, an increased *g*_m_ in response to increased [CO_2_] in the present study (Fig. 7e) suggested that, in *T. dentata*, other mechanisms may be involved. Terashima et al. (2011) suggested that porosity and tortuosity of the cell wall, or diffusion of HCO_3_^−^ could be affected by pH via changes in [CO_2_], and thus affect CO_2_ diffusion through cell walls. Irrespective of the mechanisms for *g*_m_, *g*_m_ imposes major photosynthetic limitations in ferns (Carriquí et al. 2015). This is a major reason why the *g*_m_ response of ferns to [CO_2_] has a large effect on the response of photosynthesis (*A*) to [CO_2_] (Fig. 7 g–i). A decrease in *g*_m_ in response to low [CO_2_] is clearly not advantageous for photosynthesis in the present low atmospheric CO_2_ environment because low *g*_m_ (Fig. 7d) causes low *C*_c_ (Fig. 7 m) and thus a diminished rate of photosynthesis (Fig. 7). The regulation mechanism of *g*_m_ with changes in [CO_2_] may have developed with plant evolution in response to historical changes in atmospheric CO_2_ levels and *g*_s_.

## Conclusion

In a steady-state with the present level of CO_2_ in the environment (400 μmol mol^−1^), two ferns *P.* *aquilinum* and *T. dentata* had lower mesophyll conductance (*g*_m_) than the angiosperms measured so far, which may be partly imposed by the small *S*_c_ observed. The dynamic response of *g*_s_ to changes in [CO_2_] confirmed previous studies that reported slight increases in *g*_s_ after changes in [CO_2_] from the present level to below the present level (e.g., 200 μmol mol^−1^) for fern species, in which the sensitivity of *g*_s_ to the decrease in [CO_2_] was much lower than in angiosperms. Although a causal mechanism for the CO_2_ response of *g*_m_ still remains to be clarified, the dynamic response in *g*_m_ showed a rapid and significant decrease to decreased [CO_2_] (from 400 to 200 μmol mol^−1^), which was in contrast to the response of angiosperms. These dynamic CO_2_ responses in *g*_s_ and *g*_m_ in *P. aquilinum* and *T. dentata* suggest that fern species have not evolved efficient regulatory mechanisms to cope with the low CO_2_ levels. Future studies of the CO_2_ response of *g*_m_ and *g*_s_ in ferns, and studies of *g*_m_ in other primitive plants, such as mosses, will be helpful to further elucidate how photosynthetic traits have evolved in the history of land plants.
